# Physicochemical characteristics and anti-colorectal cancer activity of *Salvia miltiorrhiza* Bunge polysaccharides

**DOI:** 10.3389/fnut.2026.1797015

**Published:** 2026-04-08

**Authors:** Xinran Wang, Jingjie Xu, Zhijun Liu, Kenan Huang, Fengjing Xu, Xinhua Song, Jianpeng Jiao, Shouhong Gao, Wansheng Chen

**Affiliations:** 1 Department of Pharmacy, Second Affiliated Hospital of Naval Medical University, Shanghai, China; 2 Research and Development Center of Chinese Medicine Resources and Biotechnology, Institute of Chinese Materia Medica, Shanghai University of Traditional Chinese Medicine, Shanghai, China; 3 Department of Thoracic Surgery, Shanghai Changzheng Hospital, Navy Military Medical University, Shanghai, China; 4 Department of Traditional Chinese Medicine, Shanghai Changzheng Hospital, Navy Medical University, Shanghai, China

**Keywords:** apoptosis, colorectal cancer, combined effect, regorafenib, *Salvia miltiorrhiza* polysaccharide, STAT3 signaling

## Abstract

**Introduction:**

*Salvia miltiorrhiza* (*S. miltiorrhiza*) Bunge is a traditional, medicinal and edible plant with diverse biological properties.

**Methods:**

In this study, a novel acidic polysaccharide (SMP-3) was extracted from *S. miltiorrhiza* roots using hot water extraction, followed by purification through DEAE-52 cellulose and Sephadex G-100 column chromatography. Structural characterization was performed utilizing FT-IR, NMR, XRD, and CD spectroscopy. The anti-colorectal cancer activity and underlying mechanisms of SMP-3, both alone and in combination with regorafenib (REG), were evaluated in vitro using HCT116 colorectal cancer cells and normal FHC cells through cytotoxicity, Western blot, and immunofluorescence analyses.

**Results:**

The purified SMP-3 exhibited a total polysaccharide content of 96.52% with a weight-average molecular weight of 61.3 kDa. Monosaccharide composition analysis revealed that SMP-3 was primarily composed of galacturonic acid, glucuronic acid, glucose, mannose, galactose, and rhamnose in a molar ratio of 52.6:24.1:7.5:6.8:6.0:3.0, preliminarily suggesting a possible pectin-like structural feature with uronic acids accounting for 76.7% of the total content. Structural characterization indicated the presence of both α- and β-glycosidic linkages, an amorphous structure, and an ordered conformation in aqueous solution. Biologically, SMP-3 exhibited dose- and time-dependent cytotoxicity against HCT116 cells while showing minimal toxicity to normal FHC cells. Notably, combination treatment with SMP-3 and REG demonstrated combined effects, significantly enhancing G0/G1 cell cycle arrest, apoptosis induction, and inhibition of cell migration and invasion compared to single-agent treatments. Mechanistically, the combination treatment upregulated p21, Bax, and caspase-3 expression while downregulating cyclin D1, CDK4, and Bcl-2, and profoundly suppressed the phosphorylation of STAT3, AKT, and ERK signaling pathways.

**Discussion:**

These findings suggest that SMP-3 possesses distinct structural characteristics and significant anti-tumor efficacy, making it a promising medical food candidate for colorectal cancer treatment.

## Graphical Abstract

**Figure:**
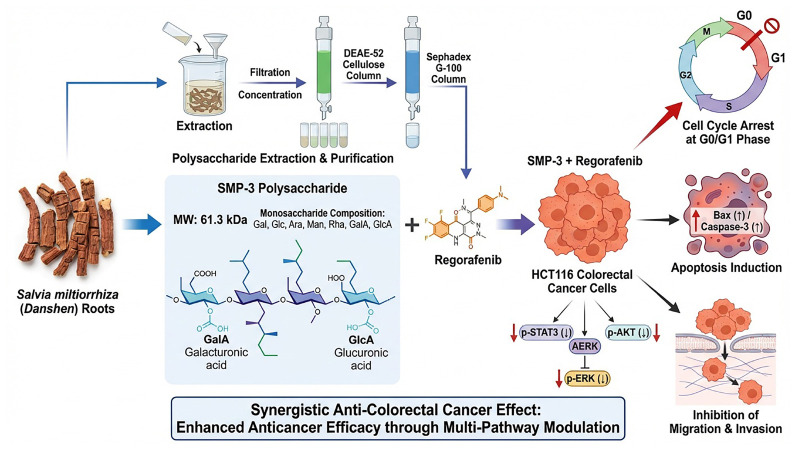

## 1 Introduction

Colorectal cancer (CRC) ranks among the most prevalent malignancies worldwide, with rising incidence rates particularly in developing countries (1). Despite advances in therapeutic strategies, drug resistance remains a major obstacle in CRC treatment. Regorafenib, an oral multi-kinase inhibitor approved for metastatic CRC, targets various signaling pathways involved in tumor growth and angiogenesis (2). However, its clinical efficacy is often limited by acquired resistance and dose-limiting toxicities (3). This situation has prompted researchers to explore combination strategies that may enhance drug sensitivity while reducing adverse effects.

Natural polysaccharides derived from traditional medicinal and edible plants have attracted considerable attention in cancer research due to their diverse biological activities and favorable safety profiles (4, 5). These macromolecules exhibit immunomodulatory, antioxidant, and direct antitumor properties through multiple mechanisms (6). Importantly, polysaccharides from various sources have demonstrated the ability to sensitize cancer cells to conventional chemotherapeutic agents (7).

*S. miltiorrhiza* Bunge, commonly known as Danshen, is a traditional Chinese medicinal herb that has been used for centuries to treat cardiovascular and cerebrovascular diseases (8). The root of this plant contains numerous bioactive compounds, including lipophilic diterpenoids, hydrophilic phenolic acids, and polysaccharides (9). While the pharmacological effects of tanshinones and salvianolic acids have been extensively studied, the polysaccharide fraction has received relatively less attention. Recent studies have shown that *S. miltiorrhiza* polysaccharides (SMPs) possess antioxidant, immunomodulatory, and antitumor activities (10). The structural features of these polysaccharides, including their monosaccharide composition, molecular weight, and glycosidic linkages, are closely related to their biological functions (11).

The relationship between polysaccharide structure and bioactivity has become a central focus in functional food research (12). Acidic polysaccharides containing uronic acid residues often exhibit enhanced biological activities compared to neutral polysaccharides (13). Moreover, the molecular weight of polysaccharides influences their membrane permeability and cellular uptake, thereby affecting their pharmacological efficacy (14). Understanding these structure-activity relationships is essential for developing polysaccharide-based nutraceuticals and functional foods with targeted health benefits.

The present study aimed to extract, purify, and characterize polysaccharides from *S. miltiorrhiza* Bunge, and to investigate their potential to enhance the anticancer activity of regorafenib against colorectal cancer cells. We hypothesized that SMPs might sensitize CRC cells to regorafenib treatment through modulation of apoptosis and survival signaling pathways. The findings from this research may provide scientific evidence for the development of SMP-based functional foods or dietary supplements as adjuvant therapies for CRC patients.

## 2 Materials and methods

### 2.1 Materials and reagents

Dried roots of *S. miltiorrhiza* Bunge were purchased from a licensed traditional Chinese medicine supplier (Shanghai, China). Human colon adenocarcinoma HCT116 and human normal colorectal mucosal FHC cells were obtained from the Cell Bank of the Chinese Academy of Sciences (Shanghai, China). Regorafenib was obtained from Selleck Chemicals (Houston, TX, United States). DEAE-52 cellulose and Sephadex G-100 were purchased from GE Co. (Cudahy, WI, United States). Standard monosaccharides including Fucose (Fuc), galactosamine hydrochloride (GalN), rhamnose (Rha), arabinose (Ara), glucosamine hydrochloride (GlcN), galactose (Gal), glucose (Glc), xylose (Xyl), mannose (Man), fructose (Fru), ribose (Rib), galacturonic acid (GalA), guluronic acid (GulA), glucuronic acid (GlcA), mannuronic acid (ManA) were obtained from Shanghai yuanye Bio-Technology Co., Ltd. (Shanghai, China). The human colorectal cancer cell line HCT116 was obtained from the Cell Bank of the Chinese Academy of Sciences (Shanghai, China). The human normal colorectal mucosal cell line FHC was obtained from Shanghai Binsui Biotechnology Co., Ltd. (Shanghai, China). DMEM medium, fetal bovine serum (FBS), and penicillin-streptomycin were purchased from Gibco (Carlsbad, CA, United States). The Cell Counting Kit-8 (CCK-8) was obtained from Solarbio (Beijing, China). Annexin V-FITC/PI apoptosis detection kit was purchased from BD Biosciences (San Jose, CA, United States). Primary antibodies against B-cell lymphoma 2 (Bcl-2), Bcl-2-associated X protein (Bax), cleaved caspase-3, cleaved poly (ADP-ribose) polymerase (PARP), signal transducer and activator of transcription 3 (STAT3), phosphorylated-STAT3 (p-STAT3), protein kinase B (AKT), phosphorylated-AKT (p-AKT), extracellular signal-regulated kinase (ERK), phosphorylated-ERK (p-ERK), and β-actin were obtained from Cell Signaling Technology (Danvers, MA, United States). All other chemicals and solvents were of analytical grade.

### 2.2 Extraction of crude polysaccharides

The dried *S. miltiorrhiza* roots were washed, oven-dried at 60°C, and ground to pass through a 60-mesh sieve. The powder was stored in a desiccator until use. Five hundred grams of the powder were extracted with distilled water at a solid-to-liquid ratio of 1:20 (w/v) in a water bath at 85°C for 3 h. A solid-to-liquid ratio of 1:20 was selected to ensure complete swelling of the plant matrix and to prevent excessive viscosity of the extraction system, thereby facilitating mass transfer and subsequent filtration. The extraction was repeated three times, and the combined extracts were centrifuged at 3,500 rpm for 15 min to remove residues. The supernatant was concentrated to one-fifth of its original volume using a rotary evaporator under reduced pressure. Four volumes of 95% ethanol were added to the concentrate, and the mixture was kept at 4°C for 24 h to precipitate polysaccharides. The precipitate was collected by centrifugation, washed sequentially with anhydrous ethanol, acetone, and diethyl ether, and then freeze-dried to obtain crude polysaccharides.

### 2.3 Deproteinization

Protein removal was performed using the Sevag method combined with enzymatic treatment. The crude polysaccharides were dissolved in distilled water at a concentration of 5 mg/mL. One-fifth volume of Sevag reagent (chloroform:n-butanol = 4:1, v/v) was added, and the mixture was vigorously shaken for 30 min. After centrifugation, the denatured protein at the interface was removed. This procedure was repeated five times. Papain (enzyme activity ≥ 3,000 U/mg) was then added to the solution, and enzymatic hydrolysis was performed at 55°C and pH 6.5 for 2 h. The enzyme was inactivated by heating at 100°C for 10 min. The solution was dialyzed against distilled water using a dialysis membrane with a molecular weight cut-off of 3.5 kDa for 48 h. The dialysate was freeze-dried to obtain deproteinized polysaccharides.

### 2.4 Purification of polysaccharides

The deproteinized polysaccharides were dissolved in distilled water at 20 mg/mL and loaded onto a DEAE-52 cellulose column (5 × 60 cm) that had been pre-equilibrated with distilled water. The column was eluted stepwise with distilled water, 0.1 M, 0.3 M, and 0.5 M NaCl solutions at a flow rate of 2 mL/min. Fractions of 10 mL were collected, and the polysaccharide content in each fraction was determined by the phenol-sulfuric acid method (14). Fractions corresponding to the major peaks (namlely SMP-1, SMP-2, SMP-3, and SMP-4) were pooled, dialyzed, concentrated, and freeze-dried. The main fraction SMP-3 obtained from DEAE-52 chromatography was dissolved in 0.3 M NaCl at 10 mg/mL and applied to a Sephadex G-100 column (2.6 × 100 cm). The column was eluted with 0.3 M NaCl at a flow rate of 0.5 mL/min. Fractions were monitored by the phenol-sulfuric acid method and UV absorbance at 280 nm. The fraction showing a single symmetrical peak was collected, dialyzed against distilled water, and freeze-dried to obtain purified SMP-3.

### 2.5 Chemical composition analysis

The neutral polysaccharide content was determined using the anthrone-sulfuric acid method (15). The uronic acid content was measured by the meta-hydroxydiphenyl method using galacturonic acid as the standard (16). The protein content was estimated by the Bradford method using bovine serum albumin as the standard (17).

### 2.6 Molecular weight determination

The molecular weight of SMP-3 were determined using a Waters gel permeation chromatography (GPC) system equipped with a refractive index (RI) detector. An injection volume of 10.00 μL and a run time of 15.00 min were employed for each analysis. Molecular weight parameters were calculated using a broad unknown relative calibration method with dextran standards. The chromatographic data were processed using Waters Empower software, which divided the chromatogram into 100 slices across the elution volume range to calculate the differential weight fraction [dwt/d(logM)] and cumulative percentage for each slice. The weight-average molecular weight (Mw) was determined from the distribution analysis.

### 2.7 Monosaccharide composition analysis

The monosaccharide composition of SMP-3 was determined by high-performance anion-exchange chromatography (HPAEC) coupled with an electrochemical detector (ED) using a ThermoFisher ICS5000 ion chromatography system. Briefly, 5 mg of sample was precisely weighed and placed in an ampoule, then hydrolyzed with 2 mL of 3 M trifluoroacetic acid (TFA) at 120°C for 3 h. The hydrolysate was transferred to a tube and evaporated to dryness under nitrogen, reconstituted in 5 mL of deionized water with vortex mixing, and 25 μL of the solution was diluted with 975 μL of deionized water. The diluted sample was centrifuged at 12,000 rpm for 5 min, and the supernatant was filtered through a 0.22 μm microporous membrane before analysis. Chromatographic separation was performed on a Dionex CarboPac PA20 column (3 mm × 150 mm) at 30°C with a flow rate of 0.3 mL/min and an injection volume of 25 μL. The mobile phase consisted of three eluents: A (H2 O), B (15 mM NaOH), and C (15 mM NaOH containing 100 mM NaOAc), with gradient elution programed as follows: 0–18 min, 98.8% A/1.2% B/0% C; 18–20 min, linear gradient to 50% A/50% B/0% C; 20–30 min, isocratic at 50% A/50% B/0% C; 30–30.1 min, step to 0% A/0% B/100% C; 30.1–46 min, isocratic at 100% C; 46–46.1 min, step to 0% A/100% B/0% C; 46.1–50 min, isocratic at 100% B; 50–50.1 min, step to 98.8% A/1.2% B/0% C; 50.1–80 min, column re-equilibration. Monosaccharide standards were subjected to identical acid hydrolysis conditions and used for calibration. The monosaccharide content was calculated by comparing the peak areas of samples with those of standards at known concentrations, and the molar ratios were determined based on the molecular weight of each monosaccharide.

### 2.8 Structural characterization

#### 2.8.1 Fourier transform infrared spectroscopy

FT-IR spectra were recorded on a Nicolet iS50 spectrometer (Thermo Fisher Scientific, Waltham, MA, United States) using the KBr pellet method. Spectra were collected in the range of 4,000–400 cm^–1^ with a resolution of 4 cm^–1^ and 32 scans.

#### 2.8.2 Nuclear magnetic resonance

NMR spectra were acquired on a Avance III HD spectrometer (Bruker, Billerica, MA, United States). Forty milligrams of SMP-3 were dissolved in 0.5 mL D_2_O and exchanged three times to remove exchangeable protons. ^1^H NMR spectra were recorded at 298 K. The lyophilized polysaccharide sample was packed into a 4 mm zirconia rotor with a Kel-F cap. The ^13^C cross-polarization magic angle spinning nuclear magnetic resonance (^13^C CP/MAS NMR) spectrum was recorded on a Bruker AVANCE III 400 MHz spectrometer.

#### 2.8.3 Scanning electron microscopy

The samples were dried to constant weight, glued, and gilded. The surface micromorphology of the polysaccharide sample was observed by scanning electron microscope (SEM, Hitachi S-4800, Tokyo, Japan).

#### 2.8.4 X-ray diffraction

XRD patterns were obtained using an X-ray diffractometer (D8 ADVANCE, Bruker, Germany) with Cu Kα radiation. Samples were scanned over the 2θ range of 5–80° at a scanning rate of 2°/min.

#### 2.8.5 Circular dichroism

CD spectra were recorded on a Jasco J-1500 spectropolarimeter (Tokyo, Japan). The sample was dissolved in water at 0.1 mg/mL, and spectra were recorded from 190 to 350 nm to analyze the ordered structure of the polysaccharide.

#### 2.8.6 Thermogravimetric analysis

The thermal stability of SMP-3 was evaluated using a thermogravimetric analyzer (TGA, NETZSCH STA 449F3, Selb, Germany). Samples were heated from 25 to 800°C at a rate of 10°C/min under nitrogen atmosphere.

### 2.9 Anti-colorectal cancer activity by cell model

#### 2.9.1 Cell culture

HCT116 and FHC cell lines were cultured in DMEM supplemented with 10% FBS, 100 U/mL penicillin, and 100 μg/mL streptomycin at 37°C in a humidified atmosphere containing 5% CO_2_. Cells were subcultured when they reached 80–90% confluence.

#### 2.9.2 Cell viability assay

Cell viability was assessed using the CCK-8 assay. HCT116 or FHC cells were seeded in 96-well plates at a density of 5 × 10^3^ cells per well and allowed to adhere overnight. Cells were then treated with various concentrations of SMP-3 (abbreviated as SMP) (0, 25, 50, 100, 200, and 400 μg/mL), regorafenib (REG) (0, 1, 2, 4, 8, and 16 μM), or their combinations for 48 h. After treatment, 10 μL of CCK-8 solution was added to each well, and the plates were incubated at 37°C for 2 h. Absorbance was measured at 450 nm using a microplate reader. Cell viability was calculated as the percentage relative to untreated control cells.

#### 2.9.3 Colony formation assay

HCT116 cells were seeded in 6-well plates at 500 cells per well and treated with SMP (800 μg/mL), REG (4 μM), or their combination for 24 h. The medium was then replaced with fresh complete medium, and cells were cultured for 14 days. Colonies were fixed with 14% paraformaldehyde, stained with 0.1% crystal violet, and counted manually. The cell clone formation efficiency was calculated using the following formula:

Clonal formation efficiency = Average number of cell clones/Total number of cells plated (1).

#### 2.9.4 Cell cycle analysis

Cell cycle distribution was analyzed by flow cytometry using propidium iodide (PI) staining. After treatment for 48 h, cells were harvested, washed with cold PBS, and fixed with 70% ethanol at 4°C overnight. Fixed cells were washed with PBS and incubated with RNase A (100 μg/mL) and PI (50 μg/mL) at 37°C for 30 min in the dark. Cell cycle distribution was analyzed using a flow cytometer (BD FACSCalibur, San Jose, CA, United States), and data were processed using ModFit LT software.

#### 2.9.5 Apoptosis assay

Apoptosis was detected using the Annexin V-FITC/PI double staining method. HCT116 cells were treated with SMP-3, regorafenib, or their combination for 48 h. Cells were then harvested, washed with cold PBS, and resuspended in binding buffer. Annexin V-FITC and PI were added according to the manufacturer’s instructions, and samples were incubated at room temperature for 15 min in the dark. Apoptotic cells were analyzed by flow cytometry within 1 h.

#### 2.9.6 Wound healing assay

Cell migration was assessed by the wound healing assay. HCT116 cells were seeded in 6-well plates and grown to confluence. A straight scratch was made across the cell monolayer using a 200 μL pipette tip. Cells were washed with PBS to remove debris and incubated with serum-free medium containing the indicated treatments. Images were captured at 0 and 24 h using an inverted microscope. The wound closure rate was calculated as the percentage of wound area reduction compared to the initial wound area.

#### 2.9.7 Transwell invasion assay

Cell invasion was evaluated using Transwell chambers (8 μm pore size, Corning, NY, United States) coated with Matrigel. HCT116 cells (2 × 10^5^) in serum-free medium containing the indicated treatments were added to the upper chamber. Complete medium containing 10% FBS was added to the lower chamber as a chemoattractant. After 24 h, cells on the upper surface of the membrane were removed with cotton swabs. Cells that had invaded through the Matrigel and membrane were fixed, stained with 0.1% crystal violet, and counted under a microscope in five random fields.

#### 2.9.8 Reverse transcription quantitative PCR

Total RNA was extracted using TRIzol reagent, and cDNA was synthesized using a reverse transcription kit. qPCR was performed using SYBR Green Master Mix on a real-time PCR system. The relative mRNA expression levels were calculated using the 2^–ΔΔCt^ method with GAPDH as the internal reference gene. Primer sequences are listed in Supplementary Table 1.

#### 2.9.9 Western blot analysis

Total proteins were extracted from treated cells using RIPA lysis buffer containing protease and phosphatase inhibitors. Protein concentrations were determined using the BCA protein assay kit. Equal amounts of protein (30 μg) were separated by SDS-PAGE and transferred to PVDF membranes. Membranes were blocked with 5% non-fat milk in TBST for 1 h and incubated with primary antibodies overnight at 4°C. After washing, membranes were incubated with HRP-conjugated secondary antibodies for 1 h at room temperature. Protein bands were visualized using enhanced chemiluminescence and quantified by ImageJ software. β-actin was used as the loading control.

#### 2.9.10 Immunofluorescence

HCT116 cells were seeded on glass coverslips in 24-well plates and treated as indicated. After 48 h, cells were fixed with 4% paraformaldehyde, permeabilized with 0.1% Triton X-100, and blocked with 5% BSA. Cells were then incubated with primary antibodies against p-STAT3 or Cleaved Caspase-3 overnight at 4°C, followed by incubation with fluorescent secondary antibodies. Nuclei were counterstained with DAPI. Images were captured using a confocal laser scanning microscope (Leica TCS SP8, Wetzlar, Germany).

### 2.10 Statistical analysis

All experiments were performed in triplicate, and data are presented as mean ± standard deviation (SD). Statistical analysis was performed using GraphPad Prism 9.0 software. Differences between two groups were analyzed by Student’s *t*-test, while multiple group comparisons were performed by one-way analysis of variance (ANOVA) followed by Tukey’s *post-hoc* test. A *p*-value < 0.05 was considered statistically significant.

## 3 Results and discussion

### 3.1 Extraction and purification of polysaccharide

**FIGURE 1:**
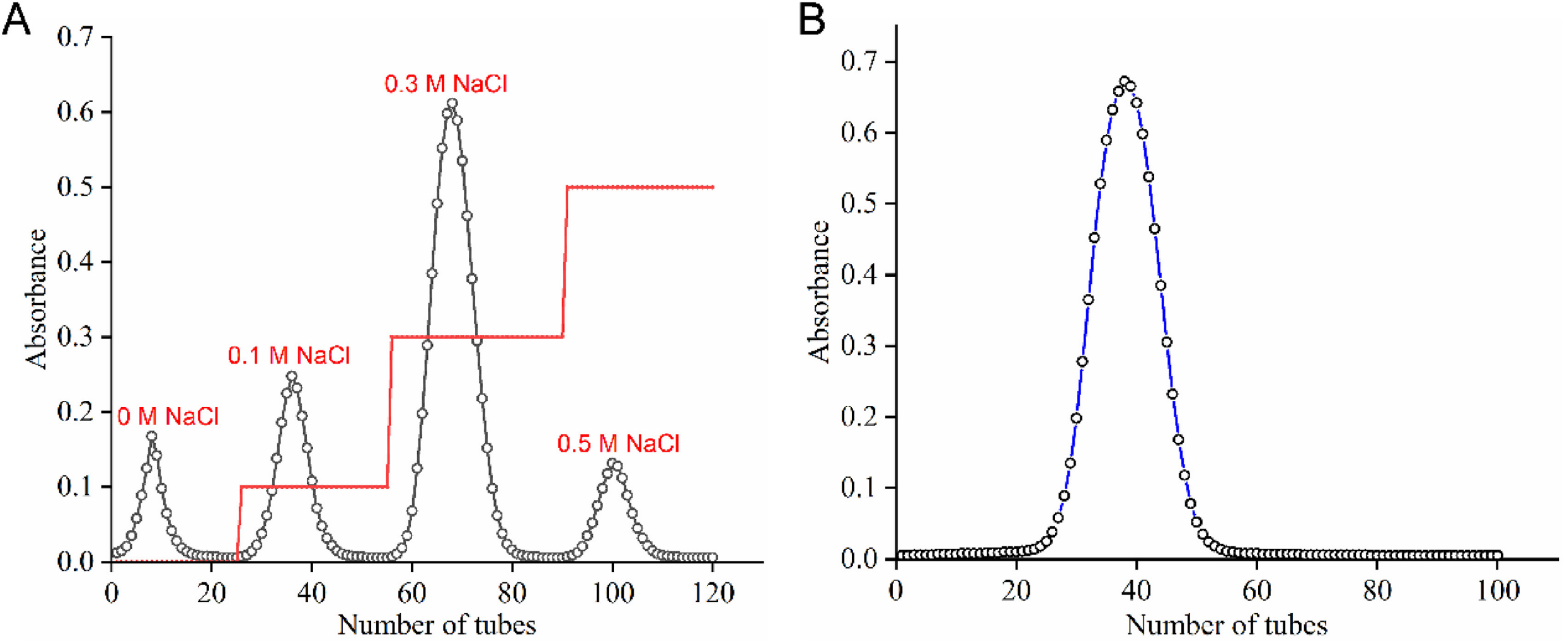
**(A)** Elution profiles of polysaccharide samples on DEAE-52 cellulose column chromatography. **(B)** Purification curves of polysaccharide samples on Sephadex G-100.

The extraction of polysaccharides from *S. miltiorrhiza* roots was performed using hot water extraction followed by ethanol precipitation. This method is widely used for polysaccharide isolation because water serves as a safe and efficient solvent for extracting hydrophilic polysaccharides (18, 19). The crude polysaccharide yield was approximately 4.5% based on the dry weight of the starting material, which is comparable to previous reports on polysaccharide extraction from medicinal plants (20). Protein removal is a critical step in polysaccharide purification, as protein contamination can interfere with structural characterization and biological activity assessment. The combination of Sevag reagent treatment and enzymatic hydrolysis effectively reduced the protein content while minimizing polysaccharide degradation. The deproteinized polysaccharides were further fractionated by DEAE-52 cellulose column chromatography. As shown in Figure 1A, the elution profile revealed four distinct fractions, designated SMP-1, SMP-2, SMP-3, and SMP-4, which were eluted with distilled water, 0.1 M, 0.3 M, and 0.5 M NaCl, respectively. The elution of polysaccharides at different salt concentrations indicates differences in their charge densities, with fractions eluted at higher salt concentrations containing more acidic groups (21). Among the four fractions, SMP-3 (eluted with 0.3 M NaCl) showed the highest yield and was selected for further purification using Sephadex G-100 gel filtration chromatography (Figure 1B).

### 3.2 Chemical composition

The chemical composition of the crude polysaccharide and purified fractions is summarized in Table 1. The crude SMP contained 45.32% total polysaccharides, 12.45% neutral polysaccharides, 32.87% acidic polysaccharides, and 8.45% protein. After ion exchange chromatography, the polysaccharide content increased substantially in all fractions. The purified SMP-3 exhibited the highest total polysaccharide content (96.52%) with only 0.62% residual protein, indicating successful purification. A notable finding was the predominance of acidic polysaccharides over neutral polysaccharides in SMP-3, with uronic acid accounting for approximately 70% of the total polysaccharide content. This high proportion of acidic residues and preliminarily suggests the possible presence of pectin-like structural features, although definitive structural assignment requires further characterization by 2D NMR spectroscopy and methylation analysis (22). Acidic polysaccharides containing galacturonic acid residues have been reported to exhibit enhanced bioactivities, including immunomodulatory and antitumor effects (23). The structural features of SMP-3 may therefore contribute to its potential health-promoting properties.

**TABLE 1:** Chemical composition of SMP fractions.

Category	Content (%)		
	Total polysaccharide	Acidic polysaccharide	Protein
SMP-1a	78.56 ± 0.98	70.33 ± 1.63	3.28 ± 0.19
SMP -2	82.34 ± 0.57	66.67 ± 0.86	2.56 ± 0.15
SMP -3	89.67 ± 1.01	61.33 ± 0.73	1.87 ± 0.12
SMP -4	75.28 ± 1.33	42.72 ± 0.37	4.35 ± 0.23

### 3.3 Molecular weight

**FIGURE 2:**
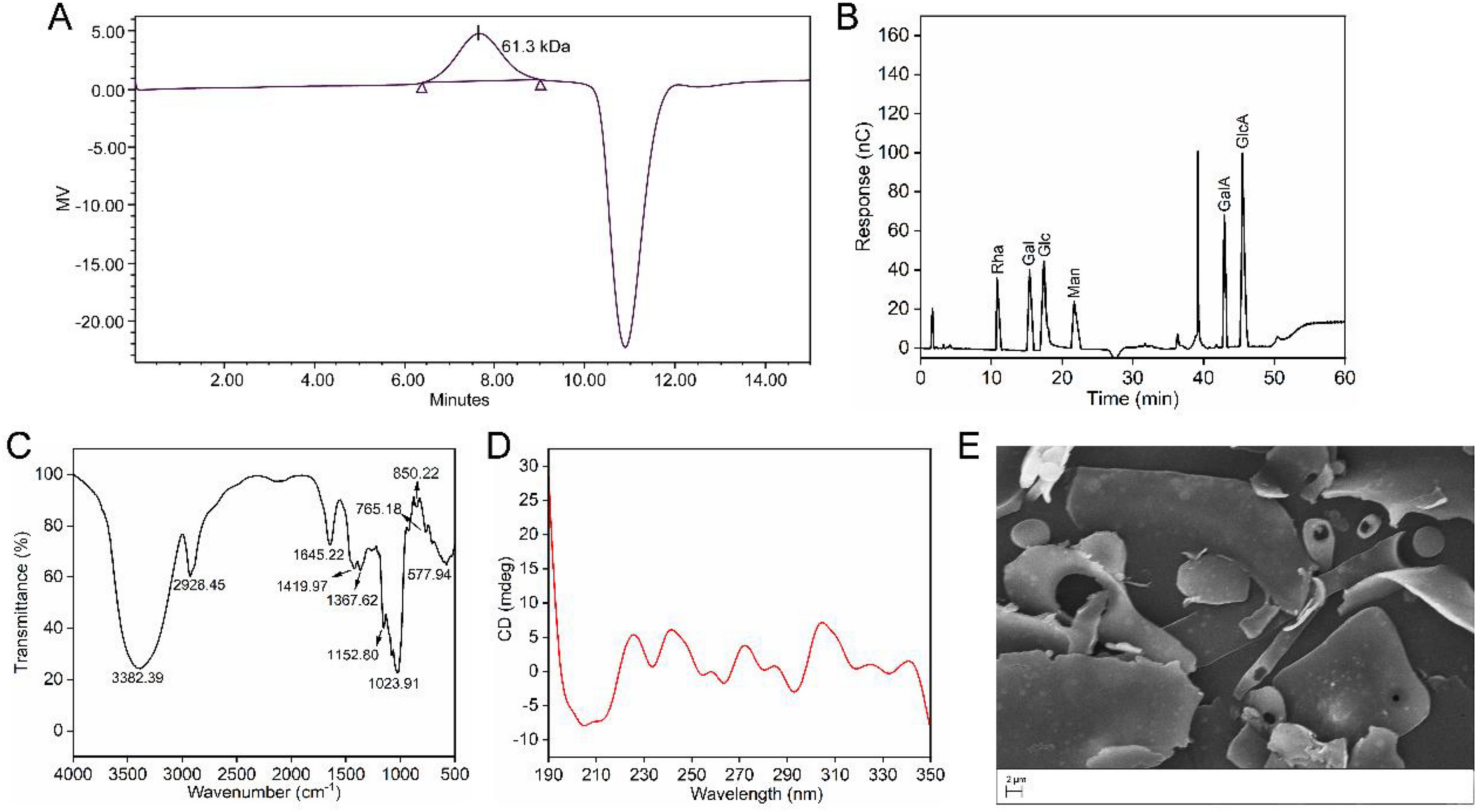
**(A)** Gel permeation chromatography profile of molecular weight. **(B)** High-performance anion-exchange chromatography of monosaccharide composition. **(C)** Fourier-transform infrared spectroscopy. **(D)** Circular dichroism. **(E)** Scanning electron microscopy analysis.

The molecular weight of SMP-3 was determined by GPC analysis. As shown in Figure 2A, SMP-3 exhibited a weight-average molecular weight (Mw) of 61.3 kDa. The molecular weight of polysaccharides is an important factor influencing their bioactivities. Polysaccharides with molecular weights in the range of 10–100 kDa have been reported to exhibit optimal bioactivity in many cases (24). The molecular weight of SMP-3 falls within this range, suggesting favorable characteristics for bioactive applications. It is worth noting that polysaccharide molecular weight can affect cellular uptake and interaction with membrane receptors, thereby influencing biological responses (22, 25).

### 3.4 Monosaccharide composition

The monosaccharide composition of SMP-3 was shown in Figure 2B, the results showed that the polysaccharide was mainly composed of galacturonic acid, glucuronic acid, glucose, mannose, galactose, and rhamnose. The molar ratios of these monosaccharides were 52.6:24.1:7.5:6.8:6.0:3.0, respectively. Uronic acids including galacturonic acid and glucuronic acid accounted for 76.7% of the total monosaccharide content, indicating that the polysaccharide was a typical acidic polysaccharide. The predominance of galacturonic acid preliminarily suggested that the polysaccharide might possess pectin-like structural features (26). However, this inference is based solely on monosaccharide composition data and needs to be confirmed by detailed linkage analysis. The presence of rhamnose together with galacturonic acid indicated the possible existence of rhamnogalacturonan domains, which is a common structural feature in plant cell wall polysaccharides and contributes to the branching architecture of pectin molecules (27). Among the neutral sugars, glucose was the most abundant component, while mannose and galactose were detected at comparable levels. These neutral sugars might form side chains attached to the acidic backbone. Previous research has demonstrated that acidic polysaccharides rich in uronic acids often possess strong antioxidant and immunoenhancing activities (28). The complex monosaccharide composition observed in this study suggested that *S. miltiorrhiza* polysaccharide might exhibit multiple pharmacological effects.

### 3.5 FT-IR spectral analysis

FT-IR spectroscopy was employed to identify the functional groups and structural features of SMP-3. The FT-IR spectrum is presented in Figure 2C. A broad and intense absorption band at 3382.39 cm^–1^ was attributed to O-H stretching vibrations, which is characteristic of polysaccharides and reflects the presence of extensive hydrogen bonding (13). The weak absorption at 2928.45 cm^–1^ was assigned to C-H stretching vibrations of methyl and methylene groups (13). Strong absorptions at 1,645 and 1,419 cm^–1^ were assigned to asymmetric and symmetric stretching vibrations of free carboxylate groups (COO^–^), respectively, confirming the presence of uronic acid moieties (14, 29). Absorption bands in the region of 1,160–1,000 cm^–1^ were attributed to C-O-C glycosidic bond vibrations and C-O stretching of pyranose rings. The absorption at 850.22 cm^–1^ indicated the presence of α-glycosidic linkages (30).

### 3.6 CD analysis

The ordered structure of SMP-3 in aqueous solution was analyzed by circular dichroism spectroscopy. The CD spectrum (Figure 2D) displayed a negative peak at approximately 210 nm, which is characteristic of carbohydrate polymers with ordered conformations. This spectral feature suggests that SMP-3 adopts a specific spatial arrangement in solution, possibly involving helical or other regular structures. The conformation of polysaccharides in solution can significantly affect their biological activities by influencing their interaction with proteins and cell surface receptors (24). The ordered structure of SMP-3 indicated by CD spectroscopy may be important for its recognition by pattern recognition receptors on immune cells and cancer cells (31).

### 3.7 Surface morphology analysis

The surface morphology of SMP-3 was examined by scanning electron microscopy at a magnification of 5,000×. The SEM image (Figure 2E) revealed that the polysaccharide exhibited an irregular flake-like or sheet-like structure with a relatively smooth surface. Some smaller fragments and rod-shaped particles were also scattered around the main structure. The smooth surface texture indicated that the polysaccharide had a compact and dense structural arrangement, which might be attributed to the strong intermolecular interactions among polysaccharide chains (32). Similar sheet-like morphologies have been reported in other plant polysaccharides, and this type of structure is often related to the aggregation behavior of polysaccharide molecules during the drying process (33). The irregular shape and non-porous surface observed in this study suggested that the polysaccharide might have limited water absorption capacity compared to polysaccharides with porous structures. The morphological characteristics of polysaccharides are known to influence their physicochemical properties and biological activities (22). The compact structure of *S. miltiorrhiza* polysaccharide observed in this study might contribute to its stability and could affect its dissolution behavior in aqueous solutions.

### 3.8 NMR analysis

**FIGURE 3:**
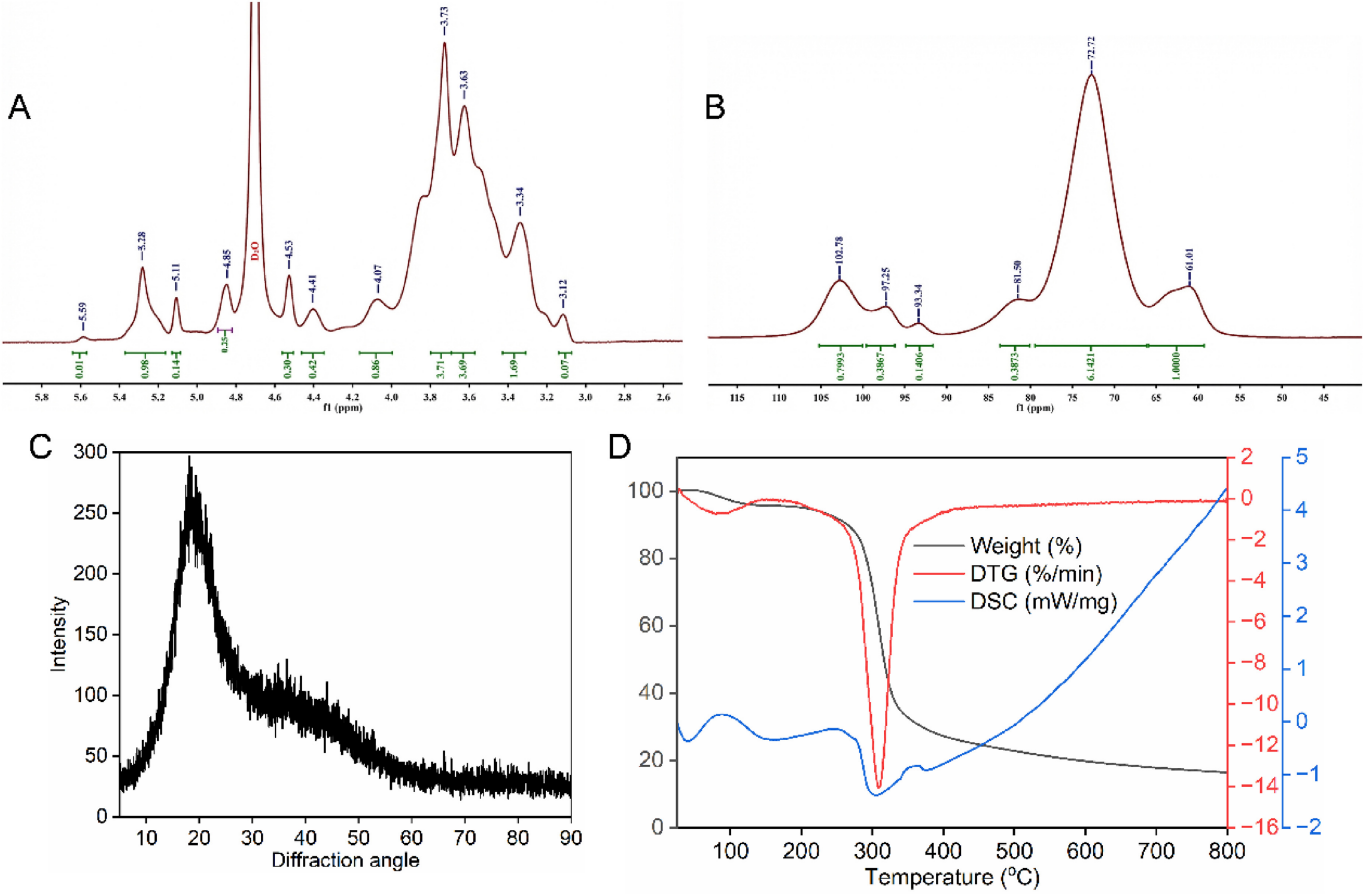
**(A)** 1H NMR spectrum. **(B)** 13C NMR spectrum. **(C)** XRD analysis. **(D)** TGA analysis.

The ^1^H NMR spectrum (Figure 3A) displayed signals mainly distributed in the region of 3.0–5.6 ppm, which is the typical chemical shift range for polysaccharides. In the anomeric region, several signals were observed at 5.59, 5.28, 5.11, and 4.85 ppm. The signal at 5.28 ppm with a relatively high intensity suggested the presence of α-configured glycosidic linkages, as α-anomeric protons typically resonate between 5.0 and 5.5 ppm (22). The signals appearing below 5.0 ppm, such as those at 4.85, 4.53, and 4.41 ppm, indicated the coexistence of β-configured sugar residues in the polysaccharide structure. The presence of both α- and β-configurations was consistent with the complex monosaccharide composition observed in the previous analysis. The strong peak at 4.70 ppm was attributed to the residual D_2_O solvent. The signals in the region of 3.12–4.07 ppm were assigned to protons at C2-C6 positions of the sugar rings. The intense peaks at 3.73 and 3.63 ppm indicated overlapping signals from multiple sugar residues, reflecting the heterogeneous nature of the polysaccharide. The solid-state ^13^C NMR spectrum (Figure 3B) provided further structural information about the polysaccharide. The anomeric carbon signals appeared in the region of 93–103 ppm. The peak at 102.78 ppm was characteristic of β-configured anomeric carbons, while the signals at 97.25 and 93.34 ppm were attributed to α-configured anomeric carbons (34). The presence of multiple anomeric carbon signals confirmed that the polysaccharide contained several types of monosaccharide residues with different glycosidic linkages. The broad and intense signal centered at 72.72 ppm corresponded to the overlapping resonances of C2, C3, and C5 carbons of pyranose rings. The signal at 81.50 ppm could be tentatively attributed to C4 carbons involved in glycosidic linkages, which may be consistent with the presence of 1,4-linked sugar residues in the polysaccharide backbone (35). The peak at 61.01 ppm was characteristic of C6 carbons in hexopyranoses, indicating that most C6 positions were unsubstituted. Combining the ^1^H and ^13^C NMR results with the monosaccharide composition analysis, it preliminarily could be concluded that *S. miltiorrhiza* polysaccharide was a heteropolysaccharide with a complex structure containing both α- and β-glycosidic linkages.

### 3.9 XRD analysis

The crystalline structure of *S. miltiorrhiza* polysaccharide was examined by X-ray diffraction analysis. The XRD pattern (Figure 3C) showed a broad diffraction peak centered at approximately 20° (2θ), which is a typical characteristic of amorphous materials. No sharp crystalline peaks were observed throughout the scanning range from 5° to 90°. This diffraction pattern indicated that *S. miltiorrhiza* polysaccharide existed predominantly in an amorphous state with a non-crystalline structure. The amorphous nature of polysaccharides is commonly observed in plant-derived polysaccharides and is attributed to the irregular arrangement of polysaccharide chains and the presence of various monosaccharide residues with different glycosidic linkages (36). The complex monosaccharide composition containing galacturonic acid, glucuronic acid, glucose, mannose, galactose, and rhamnose might contribute to the disordered molecular arrangement and prevent the formation of crystalline regions. Previous studies have demonstrated that amorphous polysaccharides generally exhibit better water solubility and bioavailability compared to crystalline polysaccharides, which could be beneficial for their biological activities (37).

### 3.10 TGA analysis

The thermal stability and degradation behavior of SMP-3 were investigated by thermogravimetric analysis coupled with differential scanning calorimetry. The result (Figure 3D) showed that the thermal degradation process occurred in three distinct stages. The first stage occurred from room temperature to approximately 200°C, during which a slight weight loss of about 5% was observed. This initial weight loss was attributed to the evaporation of free and bound water molecules in the polysaccharide sample (38). The DSC curve showed a small endothermic peak in this region, confirming the dehydration process. The second stage represented the major thermal decomposition phase, occurring between 200 and 350°C. During this stage, a significant weight loss of approximately 67.26% was observed. The DTG curve displayed a sharp peak at around 300°C, indicating the maximum decomposition rate at this temperature. This rapid weight loss was mainly caused by the cleavage of glycosidic bonds, depolymerization of polysaccharide chains, and decomposition of sugar ring structures (39). The DSC curve showed a corresponding exothermic behavior in this temperature range, reflecting the oxidative decomposition of organic matter. The third stage occurred above 350°C, where the weight loss became gradual and slow. The remaining residue at 800°C was approximately 16.45% of the original weight, which might be attributed to the formation of carbonaceous residues and inorganic ash. The thermal analysis results indicated that SMP-3 possessed good thermal stability below 200°C, which is important for its processing and storage in practical applications.

### 3.11 Anti-colorectal cancer activity

**FIGURE 4:**
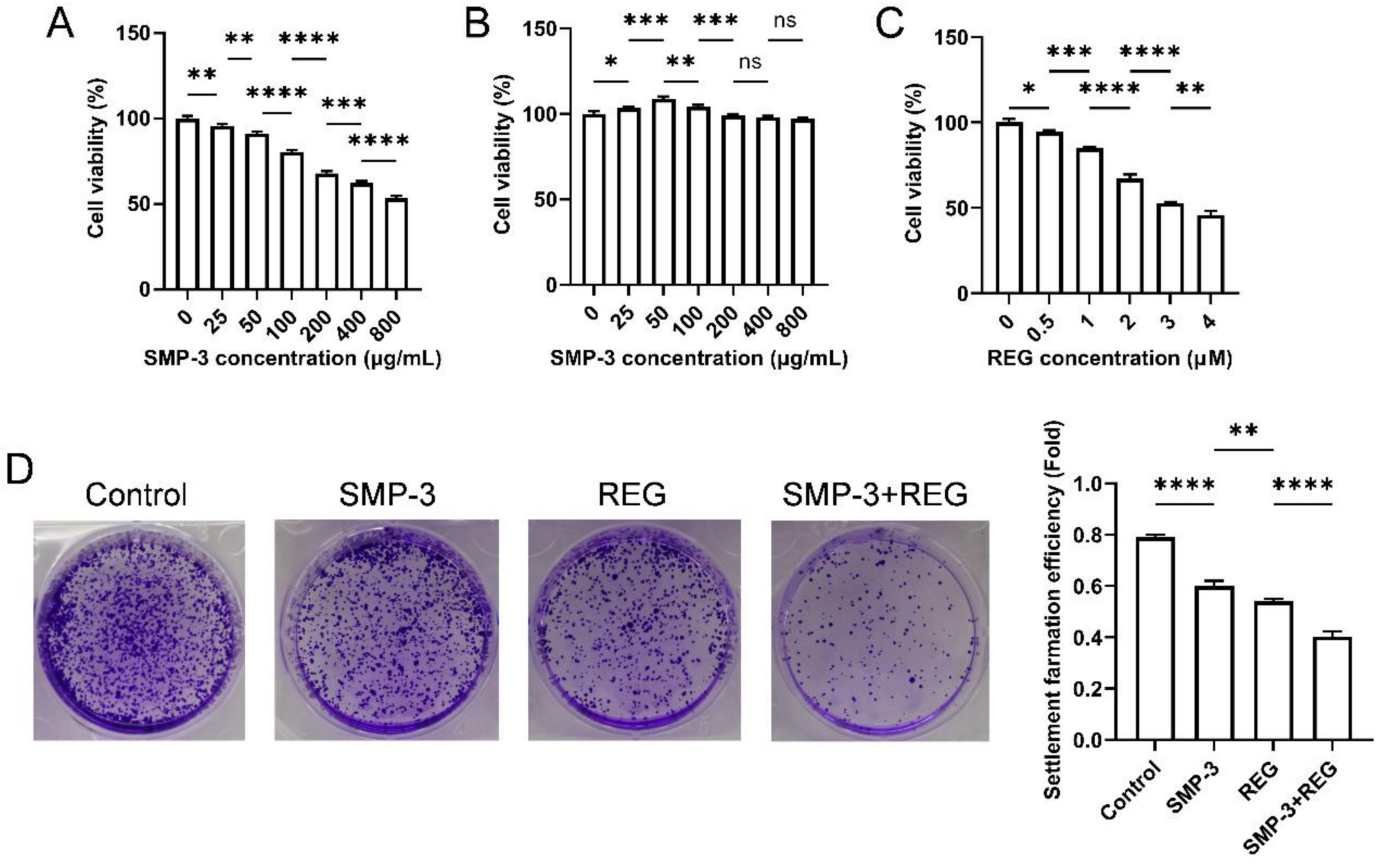
**(A)** Cell viability of HCT116 cells treated with SMP. **(B)** Cell viability of FHC cells treated with SMP. **(C)** Cell viability of HCT116 cells treated with REG. **(D)** Colony formation of HCT116 cells after treatment with SMP and REG. ****p < 0.0001; ***p < 0.001; **p < 0.01; *p < 0.05; ns, not significant.

To evaluate the cytotoxic effect of SMP-3 on colorectal cancer cells, HCT116 cells were treated with various concentrations of SMP-3 (abbreviated as SMP). As shown in Figure 4A, SMP exhibited a dose-dependent inhibitory effect on cell viability. At lower concentrations (25–100 μg/mL), SMP showed moderate cytotoxicity, while higher concentrations (200–800 μg/mL) caused more pronounced inhibition of cell proliferation. Further analysis (Figure 4B) showed that SMP had no significant cytotoxicity to normal FHC cells. The cytotoxic effect of SMP-3 against HCT116 cells suggests that this polysaccharide possesses direct antitumor activity. Plant-derived polysaccharides can inhibit cancer cell proliferation through various mechanisms, including cell cycle arrest, induction of apoptosis, and inhibition of angiogenesis (40, 41). As expected, REG exhibited strong cytotoxicity against colorectal cancer cells in the 0–4 μM range (Figure 4B). The moderate cytotoxicity of SMP-3 at the tested concentrations makes it a suitable medical food candidate for combination therapy with conventional anticancer REG drug.

The long-term antiproliferative effect of SMP and regorafenib combination was assessed by colony formation assay. As shown in Figure 4D, control HCT116 cells formed numerous colonies after 14 days of culture. Treatment with SMP-3 or REG alone moderately reduced colony numbers. The combination of SMP-3 and REG dramatically suppressed colony formation. The colony formation assay reflects the ability of single cells to proliferate and form visible colonies, which is an important indicator of tumorigenic potential (42). The pronounced inhibitory effect of combined treatment on colony formation suggests that SMP enhances the ability of REG to suppress the clonogenic survival of colorectal cancer cells. This finding supports the potential of SMP as a sensitizing agent for REG-based therapy.

**FIGURE 5:**
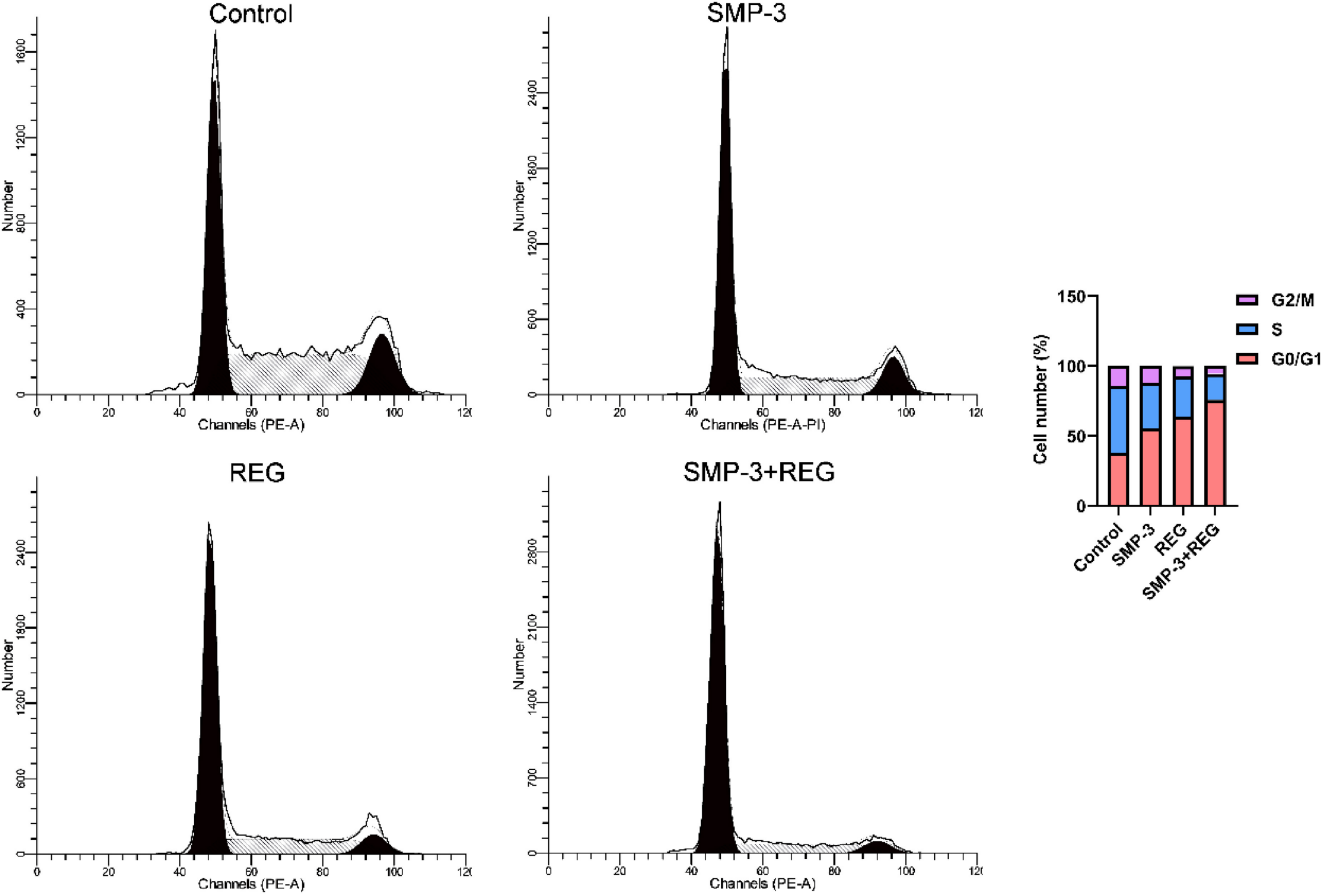
HTC116 cell cycle distribution after treatment with SMP and REG. ns, not significant.

Flow cytometric analysis was performed to examine the effect of SMP-3 and regorafenib on cell cycle progression. As shown in Figure 5, treatment with regorafenib alone induced G0/G1 arrest, with the percentage of cells in G0/G1 phase increasing from 38.26% (Control) to 63.88%. SMP treatment alone also caused moderate G0/G1 arrest (55.39%). Notably, the combination of SMP and REG produced a more pronounced G0/G1 arrest, with 75.73% of cells accumulated in this phase. The corresponding decrease in S phase and G2/M phase populations in the combination group confirms that the enhanced antiproliferative effect is associated with cell cycle inhibition. G0/G1 arrest prevents cancer cells from entering the DNA synthesis phase and ultimately undergoing mitosis. The enhanced combined effect on cell cycle arrest may involve the coordinated inhibition of cyclin-dependent kinases and other cell cycle regulatory proteins (43). These results suggest that SMP-3 enhances the cell cycle inhibitory effect of regorafenib, contributing to the observed synergy in antiproliferation.

**FIGURE 6:**
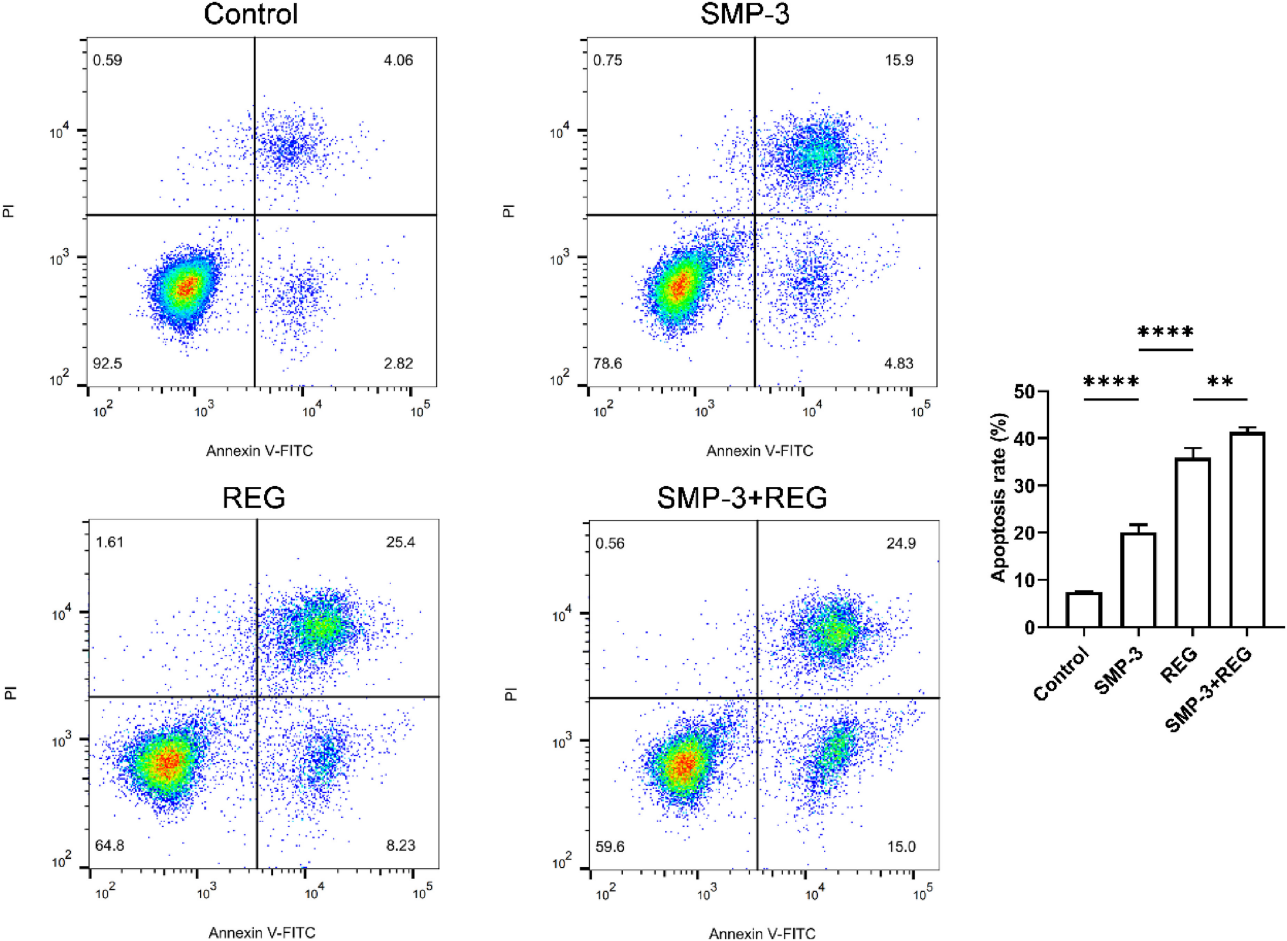
Quantification of HCT116 apoptotic cells after treatment with SMP and REG. ****p < 0.0001; **p < 0.01; ns, not significant.

The induction of apoptosis was examined using Annexin V-FITC/PI double staining. As shown in Figure 6, the total apoptotic rate (early plus late apoptosis) was significantly increased by combined treatment compared to single agent treatments. Control cells showed a basal apoptotic rate of 7.43%, which increased to 20.13% with SMP alone and 35.91% with regorafenib alone. The combination treatment resulted in a substantially higher apoptotic rate of 41.38%. The enhancement of apoptosis by combined treatment demonstrates that SMP-3 sensitizes HCT116 cells to regorafenib-induced cell death. Apoptosis is a major mechanism of cancer cell elimination by chemotherapeutic agents, and resistance to apoptosis is a hallmark of cancer (44). The ability of SMP to augment REG-induced apoptosis suggests that this polysaccharide may help overcome resistance mechanisms that limit the efficacy of regorafenib in colorectal cancer treatment.

**FIGURE 7:**
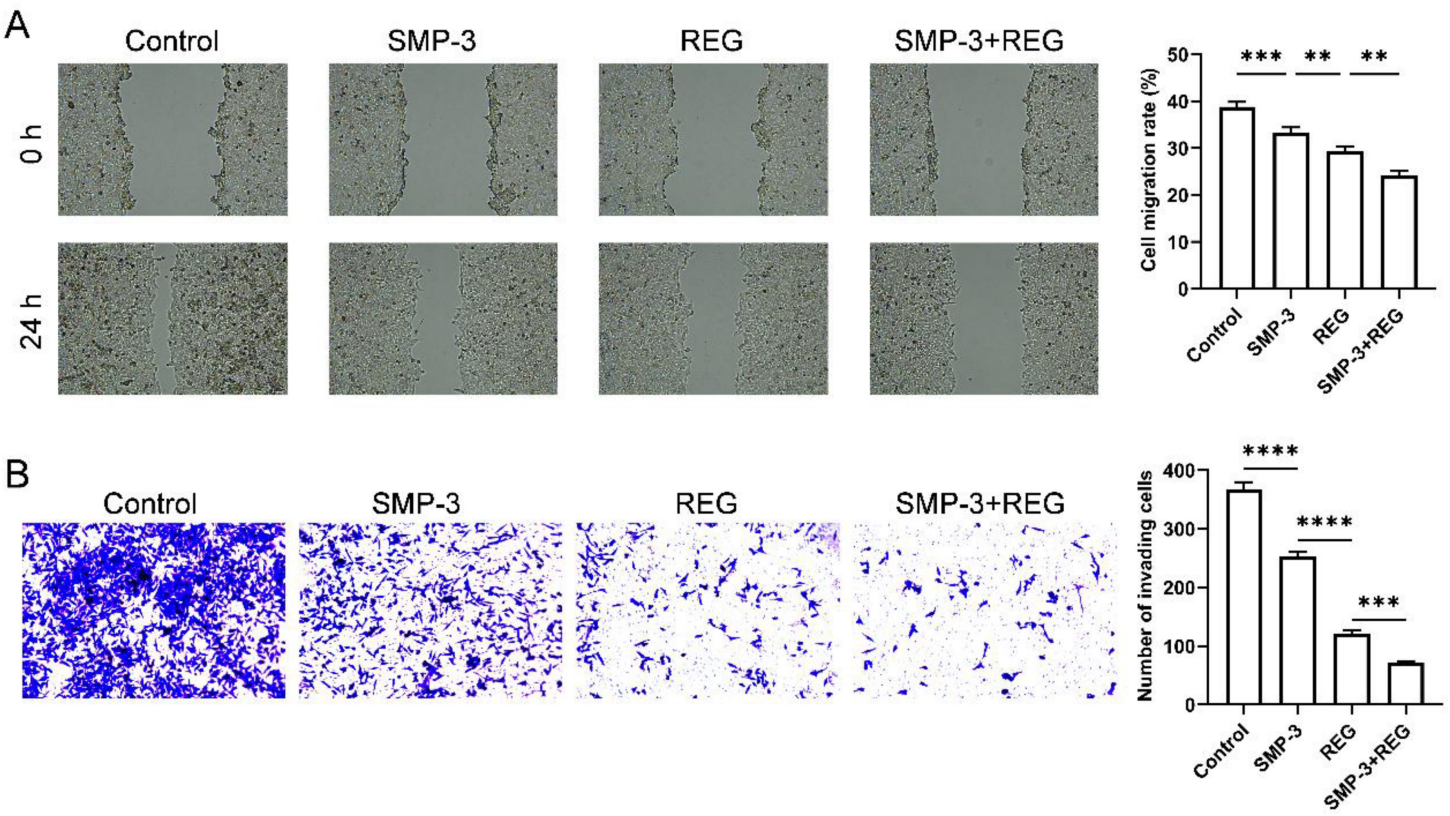
**(A)** Wound-healing assay of HCT116 cells after treatment with SMP and REG. **(B)** Transwell assay of HCT116 cells after treatment with SMP and REG. ****p < 0.0001; ***p < 0.001; **p < 0.01; ns, not significant.

The effect of SMP and regorafenib on the migratory and invasive abilities of HCT116 cells was evaluated. In the wound healing assay (Figure 7A), control cells showed 38.83% cell migration rate. SMP and REG treatment alone reduced cell migration rate to 33.28 and 29.41%, respectively. The combination treatment dramatically inhibited cell migration, with only 24.16% wound closure observed. Similar results were obtained in the Transwell invasion assay (Figure 7B). The number of invaded cells was reduced significantly following treatment with SMP and REG alone, respectively. Combined treatment resulted in a more significant reduction in invaded cell numbers compared to control. These findings indicate that SMP enhances the anti-metastatic effect of REG. Migration and invasion are critical steps in cancer metastasis, which is the leading cause of death in colorectal cancer patients (45). The ability of combined treatment to suppress both migration and invasion suggests potential benefits in preventing cancer dissemination. The anti-invasive effect may be related to the inhibition of matrix metalloproteinases or modulation of epithelial-mesenchymal transition, which warrants further investigation.

**FIGURE 8:**
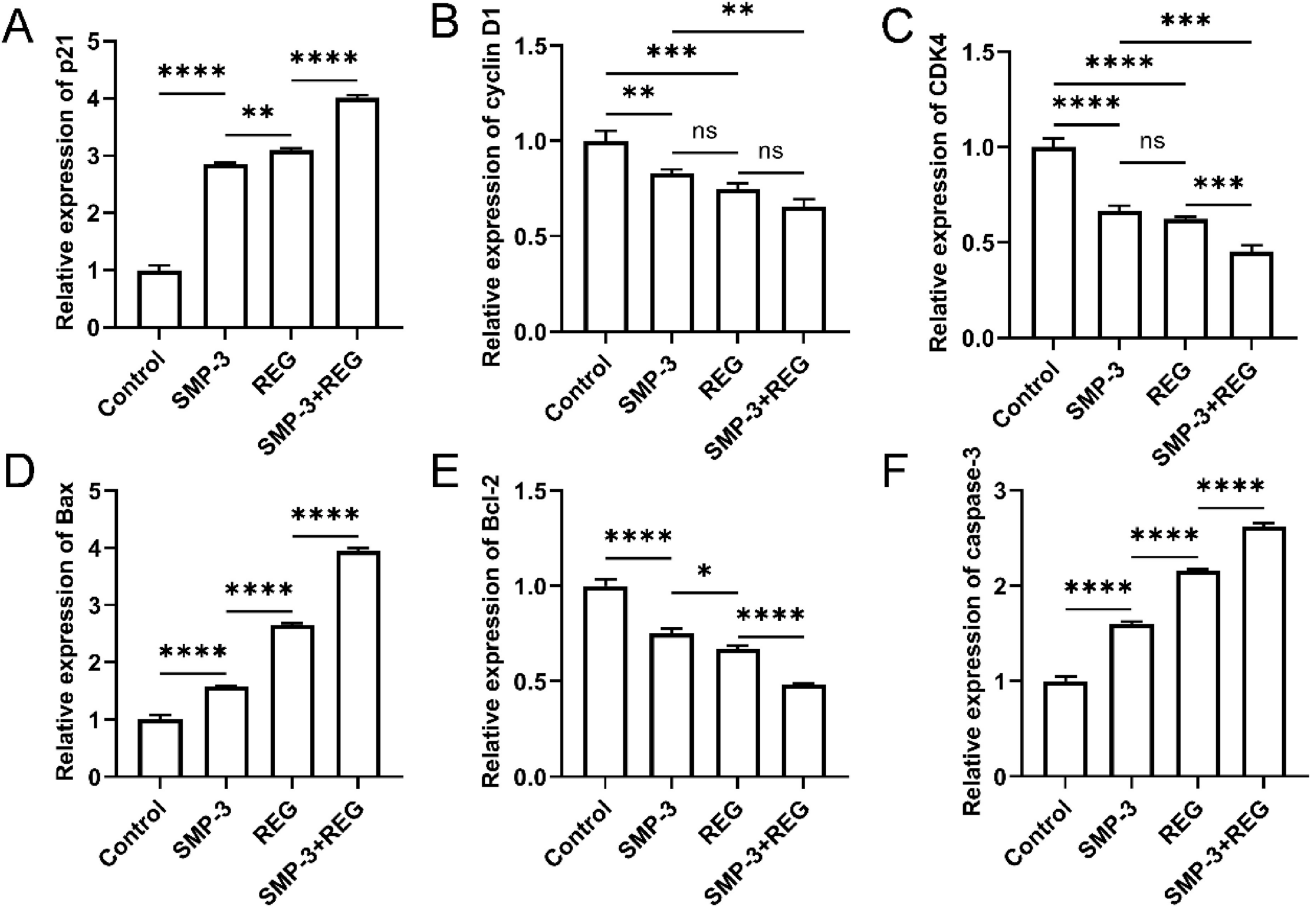
**(A–F)** Relative mRNA expression levels of cell p21 **(A)**, cyclin D1 **(B)**, CDK4 **(C)**, Bax **(D)**, Bcl-2 **(E)**, and caspase-3 **(F)** genes determined by RT-qPCR. ****p < 0.0001; ***p < 0.001; **p < 0.01; *p < 0.05; ns, not significant.

To investigate the molecular mechanisms underlying the anticancer activity of SMP against colorectal cancer cells, the expression levels of cell cycle regulators and apoptosis-related genes were examined. For cell cycle regulation, SMP treatment significantly upregulated p21 expression compared to the control group, while the combination of SMP and REG showed a synergistic effect with nearly four-fold increase (Figure 8A). Conversely, cyclin D1 and CDK4 expression were significantly downregulated following SMP treatment, and the combination treatment further suppressed CDK4 expression to approximately 40% of the control level (Figures 8B,C). These results indicated that SMP inhibited colorectal cancer cell proliferation by blocking the G1/S phase transition through modulating cell cycle regulators. For apoptosis induction, SMP treatment significantly increased Bax expression by approximately 1.5-fold, and the combination treatment resulted in a nearly four-fold elevation (Figure 8D). Meanwhile, Bcl-2 expression was decreased following SMP and REG treatments (Figure 8E). The increased Bax/Bcl-2 ratio indicated a shift toward apoptosis in colorectal cancer cells (46). Furthermore, caspase-3 expression was significantly elevated following SMP treatment by approximately 1.8-fold, and the combination treatment further enhanced it to 2.5-fold of the control level (Figure 8F), confirming that SMP induced apoptosis through the intrinsic mitochondrial pathway (47).

**FIGURE 9:**
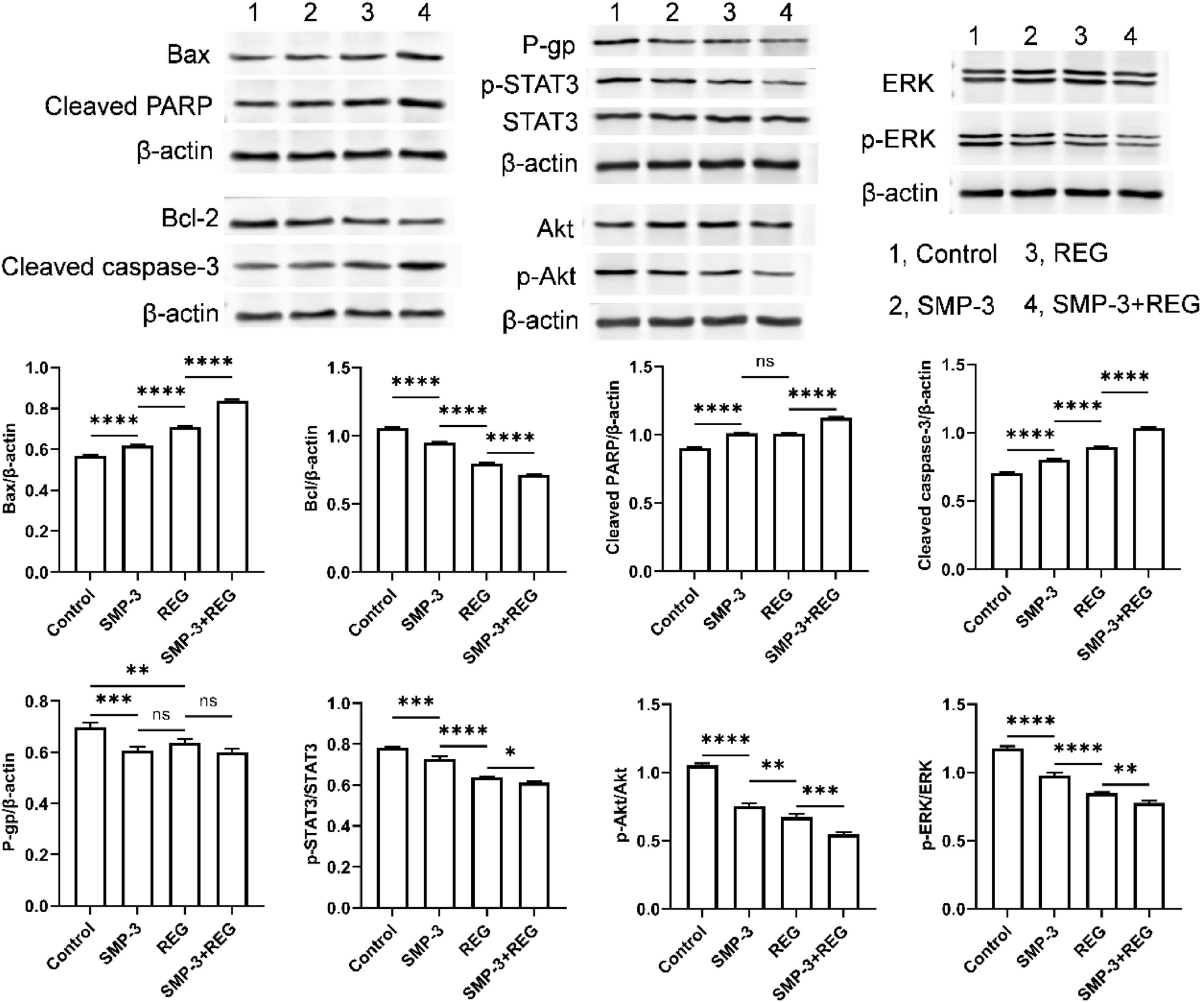
Western blot analysis of Bax, Bcl-2, cleaved PARP, cleaved caspase-3, P-gp, p-STAT3, STAT3, p-AKT, AKT, p-ERK, and ERK proteins. ****p < 0.0001; ***p < 0.001; **p < 0.01; *p < 0.05; ns, not significant.

Western blot analysis was performed to investigate the molecular mechanisms underlying the enhanced apoptotic response. As shown in Figure 9, combined treatment significantly upregulated the pro-apoptotic protein Bax while downregulating the anti-apoptotic protein Bcl-2. The Bax/Bcl-2 ratio, a critical determinant of cellular susceptibility to apoptosis, was markedly increased by combined treatment. Furthermore, the levels of cleaved caspase-3 and cleaved PARP, which are hallmarks of apoptosis execution, were substantially elevated in the combination group compared to either treatment alone. The activation of caspase-3 leads to the cleavage of PARP, a DNA repair enzyme, resulting in the irreversible commitment to apoptotic cell death (48). The pronounced activation of this apoptotic cascade by combined treatment provides a mechanistic explanation for the enhanced cytotoxic effect observed. Notably, Western blot results also revealed that the expression of P-glycoprotein (P-gp) was significantly downregulated following combined treatment with SMP-3 and regorafenib compared to the control and single-agent treatment groups (Figure 9). P-gp, encoded by the MDR1/ABCB1 gene, is a transmembrane ATP-binding cassette (ABC) transporter that actively effluxes a broad spectrum of chemotherapeutic agents out of cancer cells, thereby reducing intracellular drug accumulation and contributing to multidrug resistance (MDR) (49). In colorectal cancer, P-gp overexpression has been identified as a key mechanism underlying acquired resistance to multiple targeted therapies, including regorafenib (50). Previous studies have demonstrated that regorafenib is a substrate of P-gp, and elevated P-gp expression can significantly reduce its intracellular concentration and therapeutic efficacy (51). The observed downregulation of P-gp by SMP-3, particularly in the combination treatment group, suggests that SMP-3 may enhance the intracellular retention of regorafenib by suppressing the P-gp-mediated drug efflux mechanism. This finding provides a potential pharmacological explanation for the enhanced anticancer effects observed with the combined treatment, as increased intracellular regorafenib accumulation would be expected to strengthen the inhibition of its downstream targets, including STAT3, AKT, and ERK signaling pathways. Similar P-gp inhibitory effects have been reported for other plant-derived polysaccharides, such as Astragalus polysaccharides and Ganoderma lucidum polysaccharides, which were shown to reverse MDR in various cancer cell models (52, 53). However, the detailed molecular mechanism by which SMP-3 downregulates P-gp expression, whether through transcriptional suppression of the MDR1 gene, post-translational modification, or epigenetic regulation, remains to be elucidated in future studies.

The effect of SMP and REG on key survival signaling pathways was examined. REG treatment alone reduced the phosphorylation of STAT3, which is a known target of this multi-kinase inhibitor. Interestingly, SMP-3 also exhibited inhibitory effects on STAT3 phosphorylation. Combined treatment produced a more profound suppression of p-STAT3 levels than either agent alone. STAT3 is constitutively activated in many cancers and promotes tumor cell survival, proliferation, and resistance to apoptosis (54). The enhanced inhibition of STAT3 signaling by combined treatment may contribute to the enhanced anticancer effect. Additionally, the phosphorylation levels of AKT and ERK, which are important survival kinases, were also examined. Combined treatment showed enhanced inhibition of both p-AKT and p-ERK compared to single treatments, suggesting that SMP may help overcome compensatory activation of these survival pathways.

**FIGURE 10:**
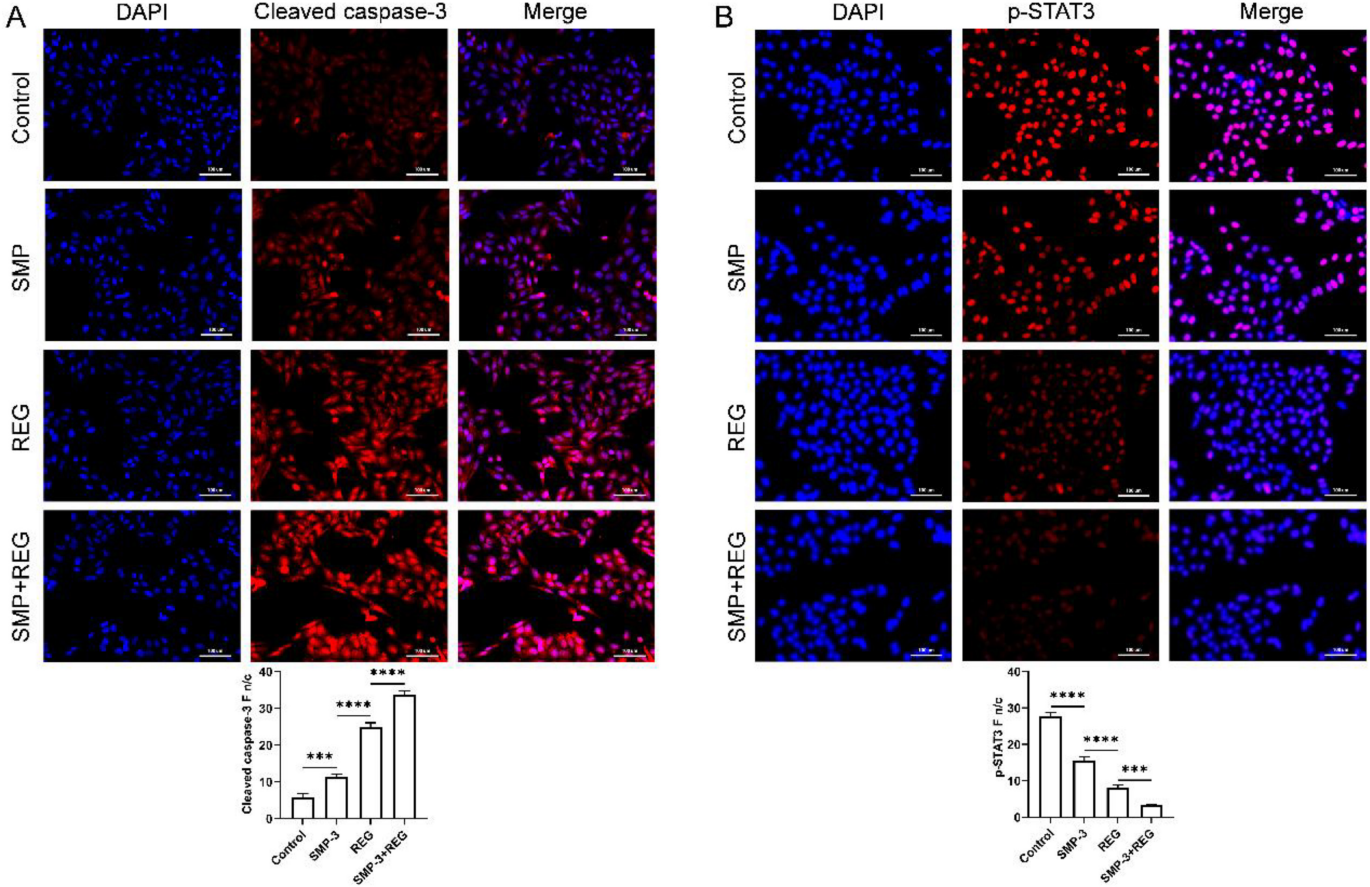
**(A,B)** Immunofluorescence assay of cleaved caspase-3 **(A)** and p-STAT-3 **(B)** proteins. ****p < 0.0001; ***p < 0.001; ns, not significant.

Immunofluorescence staining was performed to visualize the localization and expression of key proteins in treated cells. As shown in Figure 10A, cleaved caspase-3 staining was minimal in control cells but markedly increased following treatment, particularly in the combination group. The cytoplasmic distribution of cleaved caspase-3 in treated cells is consistent with its role in executing apoptosis. These immunofluorescence results corroborate the Western blot findings and provide visual confirmation of the molecular changes induced by combined treatment. In contrast, p-STAT3 was predominantly localized in the nucleus of control cells (Figure 10B), reflecting its transcriptionally active state. Treatment with SMP-3 or regorafenib alone reduced nuclear p-STAT3 staining, and combined treatment further diminished p-STAT3 fluorescence intensity. Based on our findings, we propose that SMP enhances the anticancer activity of REG through multiple complementary mechanisms. First, SMP-3 augments the inhibitory effect of REG on STAT3 signaling, leading to reduced expression of survival genes such as survivin, cyclin D1, and c-Myc. Second, SMP-3 inhibits compensatory activation of AKT and ERK pathways that may contribute to REG resistance. Third, SMP-3 shifts the Bax/Bcl-2 balance toward apoptosis and enhances caspase-mediated cell death.

The multi-target mechanism of action and the observed anti-colorectal cancer activity of SMP-3 are closely related to its structural characteristics. Firstly, SMP-3 is an acidic polysaccharide rich in uronic acids (76.7%), particularly galacturonic acid. Previous studies have demonstrated that the carboxyl groups in uronic acids can alter the charge distribution on the cancer cell membrane, facilitating the binding of polysaccharides to specific cell surface receptors and triggering intracellular apoptotic signaling pathways (55). Secondly, the weight-average molecular weight of SMP-3 (61.3 kDa) falls within the optimal range (10–100 kDa) for crossing biological barriers and exerting antitumor effects without causing severe steric hindrance (24). Furthermore, the ordered conformation indicated by CD spectroscopy may provide a specific spatial arrangement that enhances its recognition and affinity by pattern recognition receptors on HCT116 cells, ultimately leading to cell cycle arrest and apoptosis. Thus, the favorable safety profile of plant-derived polysaccharides makes SMP-3 an attractive candidate for clinical evaluation as an adjuvant medical food.

## 4 Conclusion

In conclusion, a novel acidic polysaccharide (SMP-3) was successfully extracted and purified from *S. miltiorrhiza* Bunge roots. The purified polysaccharide possessed a molecular weight of 61.3 kDa and displayed preliminary pectin-like structural features as a heteropolysaccharide with galacturonic acid as the predominant monosaccharide component. Comprehensive structural analyses confirmed its amorphous nature, ordered solution conformation, and the presence of both α- and β-glycosidic linkages. The anti-colorectal cancer investigation demonstrated that SMP-3 selectively inhibited HCT116 cancer cell proliferation without significant cytotoxicity toward normal colorectal cells. More importantly, combination therapy with SMP-3 and regorafenib exhibited enhanced combined anticancer effects, manifested by enhanced cell cycle arrest at the G0/G1 phase, increased apoptosis, and suppressed cell migration and invasion. Mechanistic studies revealed that the synergistic effect was mediated through coordinated modulation of multiple signaling pathways, including upregulation of p21 and pro-apoptotic proteins (Bax, caspase-3), downregulation of cell cycle promoters (cyclin D1, CDK4) and anti-apoptotic Bcl-2, and simultaneous inhibition of STAT3, AKT, and ERK phosphorylation. These findings provide scientific evidence supporting the potential application of *S. miltiorrhiza* polysaccharide as an adjuvant medical food to enhance the therapeutic efficacy of regorafenib in colorectal cancer treatment.

## Data Availability

The original contributions presented in this study are included in the article/Supplementary material, further inquiries can be directed to the corresponding authors.
