# Supplementary material for: Physicochemical characteristics and anti-colorectal cancer activity of Salvia miltiorrhiza Bunge polysaccharides
Source: Front Nutr. 2026 Apr 8;13:1797015. doi: 10.3389/fnut.2026.1797015 (PMC13099326; doi:10.3389/fnut.2026.1797015)
Supplement: Supplementary file 1 [file Table_1.docx]

**Table S1**. Primers used for RT-qPCR.

| Name | Sequence |
| --- | --- |
| CDK4 | Forward- GAGGCGACTGGAGGCTTTT, Reverse- GGATGTGCACAGACGTCC |
| p21 | Forward- GCCCAGTGGACAGCGAGCAG, Reverse- GCCGGCGTTTGGAGTGGTAG |
| cyclin D1 | Forward- GCTGCGAAGTGGAAACCATC, Reverse- CCTCCTTCTGCACACATTTGAA |
| Bcl-2 | Forward- TTTCTCTCTTTCGGCCGTGG, Reverse- GACATCTCCCTGTTGACGCT |
| Bax | Forward- GCTTCAGGGTTTCATCCAG, Reverse- GGCGGCAAYCAYCCYCAYG |
| caspase-3 | Forward- CTGGACTGCGCTATTGAG, Reverse- GGGTGCGGTAGAGTAAGC |
| GAPDH | Forward- GCACCGTCAAGGCTGAGAAC, Reverse- TGGTGAAGACGCCAGTGGA |
